# Point-of-care ultrasound in obstetrics and gynecology

**DOI:** 10.1007/s00404-021-05972-5

**Published:** 2021-02-08

**Authors:** Florian Recker, Eva Weber, Brigitte Strizek, Ulrich Gembruch, Susan Campbell Westerway, Christoph F. Dietrich

**Affiliations:** 1 Department of Obstetrics and Gynecology, University Hospital Bonn, Venusberg Campus 1, 53127 Bonn, Germany; 2 Charles Sturt University, Bathurst, NSW Australia; 3 Department for Internal Medicine, Clinic Beau-Site, Schänzlihalde 11, 3013 Bern, Switzerland

**Keywords:** Point-of-care ultrasound (POCUS), Gynecology, Obstetrics, Imaging

## Abstract

**Background:**

The rapid technical development and portability of ultrasound systems over recent years has had a profound impact on the area of point-of-care-ultrasound (POCUS), both in general medicine and in obstetrics and gynecology. The use of POCUS enables the clinician to perform the ultrasound scan either at the medical office or the patient’s bedside and used as an extension of the physical examination. Real-time images can immediately be correlated with the patient’s symptoms, and any changes in a (critical) patient’s condition can be more rapidly detected.

**POCUS in OBGYN:**

POCUS is also suitable for time-critical scenarios, and depending on the situation and its dynamics, the course and results of any therapy may be observed in real time. POCUS should be considered to be a routine extension of practice for most OB/GYN clinicians as it can give immediate answers to what could be life-threatening situations for the mother and/or baby. With its proven usefulness, the applications and use of POCUS should be incorporated in teaching programs for medical students, OBGYN residents and emergency physicians.

## What is POCUS?

POCUS is an examination method in which ultrasound is brought to the patient and used as an extension of the physical examination and the real-time images may immediately be correlated with the patient's symptoms [1]. It is used by various specialties in multiple situations and can be divided into three major aspects: interventional, diagnostic, and screening applications [2–4]. POCUS is different from conventional ultrasound: it is a rapid, limited study performed at the bedside for a specific diagnostic or therapeutic purpose. The study is normally performed by the same clinician who makes the treatment decisions and who has the advantage of knowing the patient’s background and symptoms [5]. POCUS images can be rapidly obtained in real time, which allows the direct correlation of ultrasound findings with the patient’s presenting symptoms and can be repeated if the patient’s condition changes dramatically. Thus, POCUS can reduce the time to diagnosis and allows a faster initiation of necessary treatment in the clinical setting [6]. It has been shown that POCUS can serve as an accurate diagnostic adjunct [7] and can support physical examinations with the potential to augment the detection of clinically important entities [8].

This immediate diagnosis is particularly relevant in obstetrics and gynecology (OBGYN), when delayed diagnosis of obstetrical or gynecological complications lead to critical outcomes for the mother and the fetus [9]. POCUS is, however, not a substitute for an in-depth prenatal or diagnostic ultrasound scan.

## What is PUM?

Currently one of the limitations of POCUS is the lack of ultrasound equipment that is easy to move around and allows clinicians to truly bring ultrasound to the patient, instead of bringing the patient to the ultrasound machine. The introduction of portable (and more affordable) ultrasound machines (PUMs) allows for access of this imaging modality to more obstetric departments and potentially even in remote and rural regions of the world.

Modern PUMs manly consist of transducers that can be connected to a smart-phone or tablet.

Publications on POCUS in OBGYN show that diagnostic findings obtained with PUMs are similar to those generated with advanced, specialized ultrasound machines [10]. These studies overlap with various studies in other medical field such as cardiology, internal medicine and emergency medicine where PUMs have shown to be of high clinical value [10]. In 2018, the World Federation of Ultrasound in Medicine and Biology (WFUMB) published a position paper in which the key fundamentals such as definitions, possible applications and safety considerations of POCUS were discussed [11].

However, there is a still great need for a comprehensive version of the application of PUMs in OBGYN to expand and improve management decisions in labor wards, primary care and in low resourced countries [12–16]. A WFUMB project showed that training midwives in performing routine and focused prenatal scans was a convenient way to cope with the small number of trained sonographers or obstetricians in low-resource countries [17].

Ultrasound is an operator-dependent imaging technology which has major implications on education and ultrasound training [11]. The literature shows that training of physicians or sonographer/midwives in the use of basic ultrasound can be effectively achieved if those chosen for training have a positive attitude [18].

With the use of telemedicine, the transmission of ultrasound images between remote regions and ultrasound centers can be simple and fast. Rapid technical development has been a leading point in recent years, especially improving medical care in remote regions. [19]. In many low-resource countries, ultrasound is mainly available in the most highly populated and developed areas, whereas the majority of the population tends to live in poorer regions and usually have little or no access to health care. Thus, it may involve long journeys to the required medical centers for the patients and the required costs for the corresponding medical examinations can be very high. Introducing the use of solar-powered and robust ultrasound devices for POCUS into the more remote areas may reduce these costs but will only be useful if it generates a good outcome for the patient at low cost.

## POCUS applications in OBGYN

POCUS in OBGYN is particularly useful in emergency situations. The literature describes high specificity and sensitivity of POCUS in OBGYN [20], but one has to keep in mind that obtaining relevant and diagnostic results is always user dependent.

**Fig. 1:**
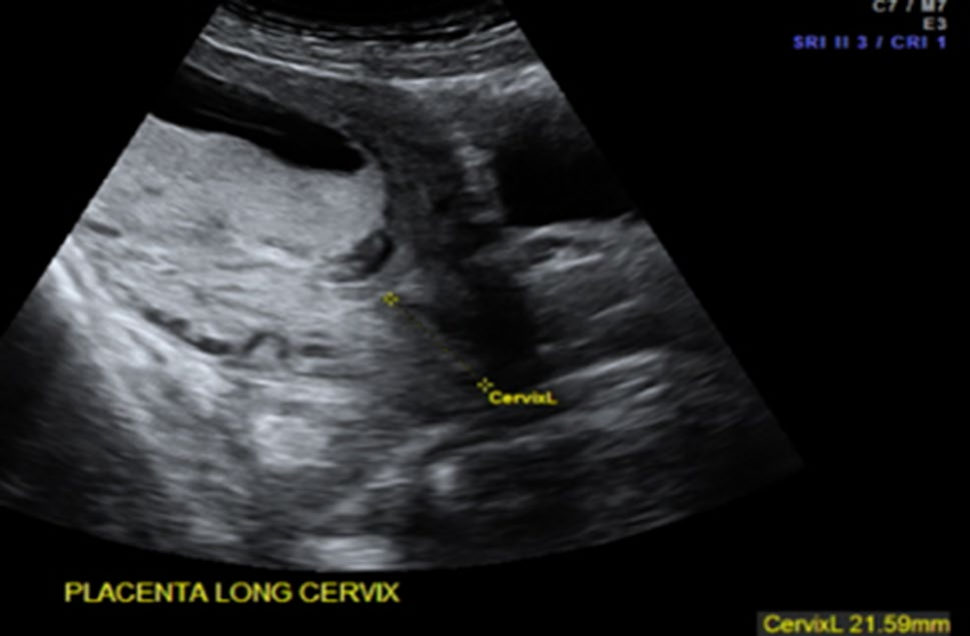
Determination of the cervical length in the second trimester (TA view)

In obstetrics, ultrasound is used to monitor the course of any pregnancy from 5 weeks of gestation to term. Midwives and obstetricians may use POCUS to confirm an intrauterine pregnancy, fetal viability, number of fetuses (twins/triplets) and gestational age. Furthermore, they can monitor the pregnancy in the second and third trimesters to assess fetal lie, fetal growth and fetal well-being as well as the placental position, measure the cervical length (Fig. 1) and determine the level of amniotic fluid that can indicate various pathological circumstances of the fetus.

Protocols for the performance of an obstetrical ultrasound depend on the qualification of the practitioner, the local legal and ethical conditions and also on the costs incurred in the examination.

**Fig. 2:**
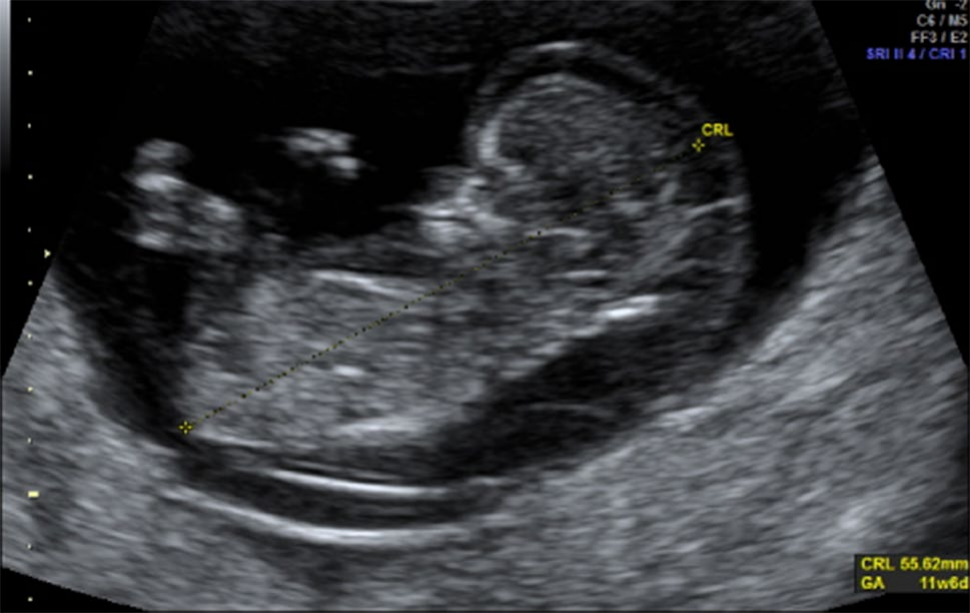
Fetus with cystic hygroma and measurement of the crown–rump length in the first trimester (TA view)

**Fig. 3:**
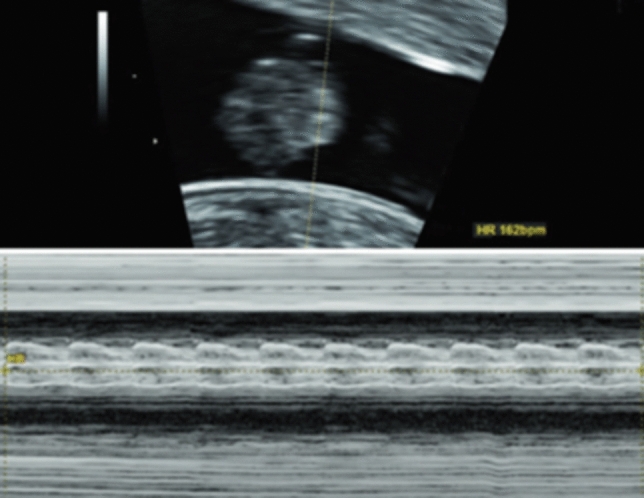
Presentation of the fetal viability in the first trimester

**Fig. 4:**
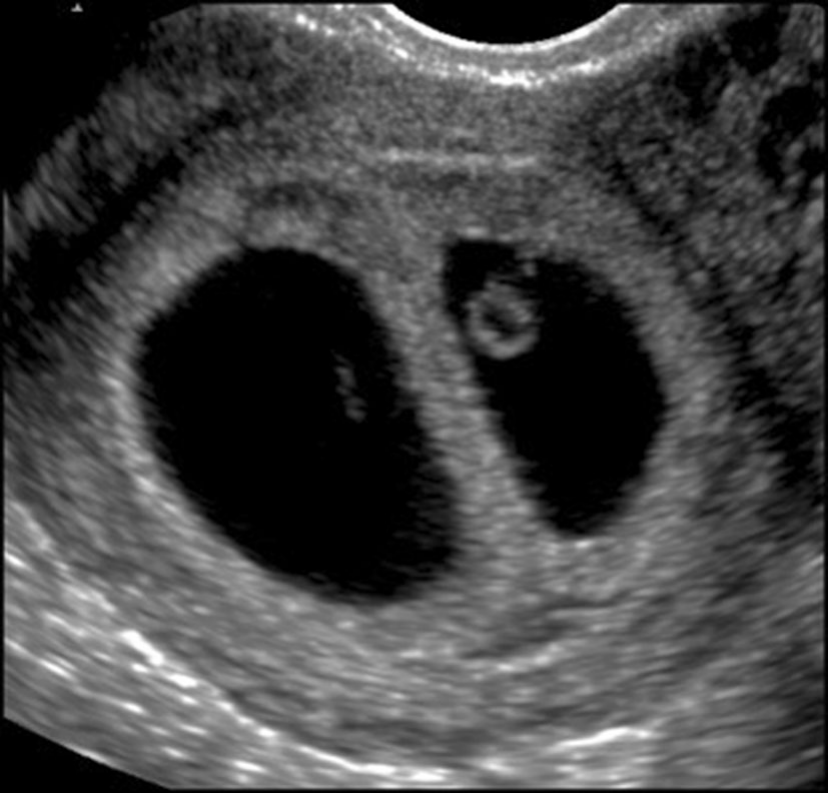
Dichorionic–diamniotic twins showing a lambda sign in the first trimester

Abdominal pain in any woman with a positive pregnancy test may be caused by an ectopic or extra-uterine pregnancy. A well-performed POCUS examination may detect a missing intrauterine gestational sac, the presence of a tubal sac or free intraperitoneal fluid due to a rupture of the fallopian tubes [21]. Findings that are relatively easy to assess include the presence and location of a gestational sac, the measurement of the crown–rump length in the first trimester (Fig. 2), fetal viability (Fig. 3), multiple pregnancy (Fig. 4), any pathological placental location such as placenta previa, abnormal amount of amniotic fluid (oligo- or polyhydramnion) or a shortening of the cervical length. All these circumstances can greatly affect both maternal and fetal morbidity and mortality, especially in low-income settings where medical care is limited.

**Fig. 5:**
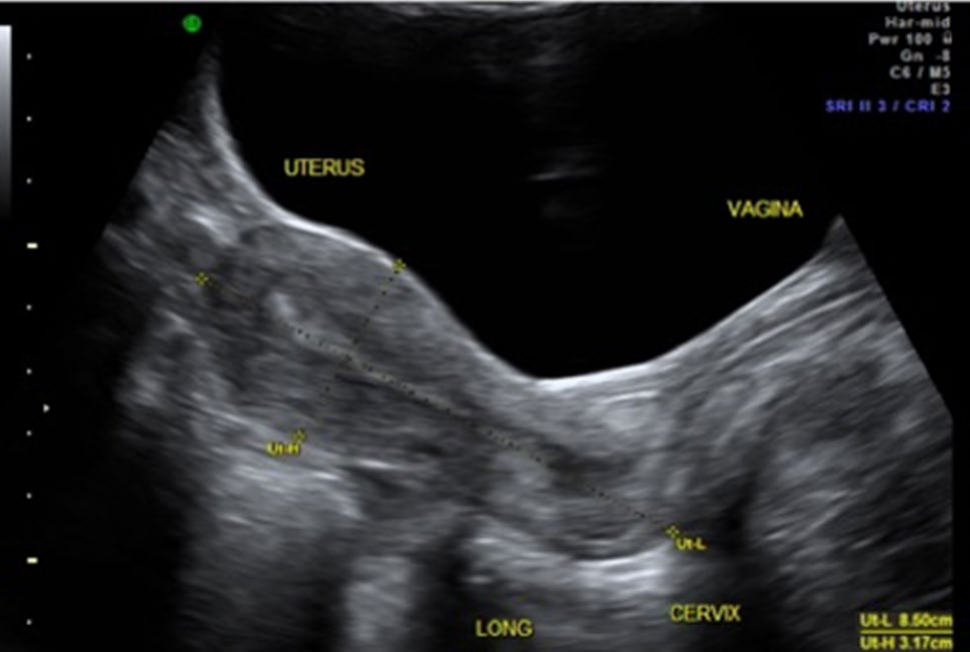
Identification of the uterine position (sagittal view from TA)

**Fig. 6:**
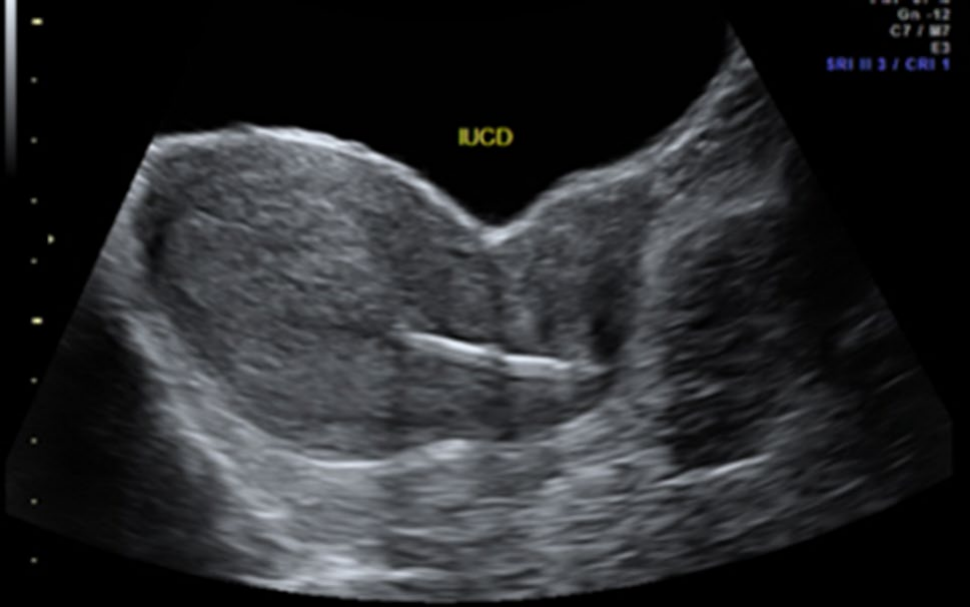
Confirmation the location of intrauterine contraceptive devices in situ (TA)

**Fig. 7:**
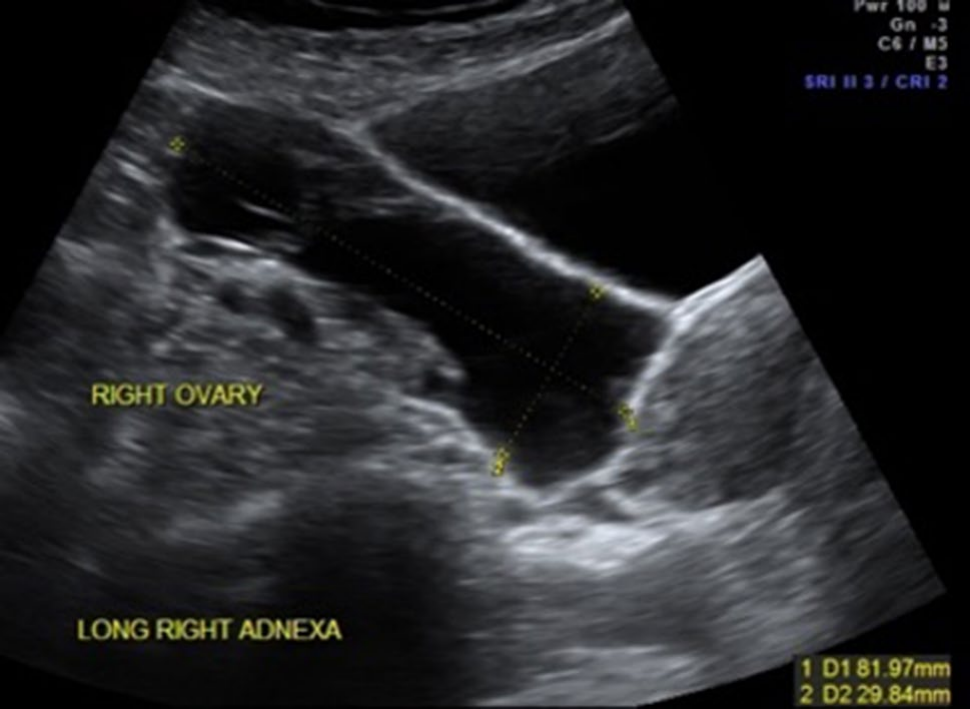
Right hydrosalpinx in a patient suffering from pain

In gynecology, POCUS can assist to identify the reason for abdominal pain, bleeding or abdominal distension or in defining a pelvic mass. It is possible to record uterine shape and position (anteverted vs retroverted) (Fig. 5), presence of fibroids, confirm the location of intrauterine contraceptive devices (Fig. 6), measure endometrial thickness, detect polyps or blood/fluid in the cavity. It is also useful to visualize ovarian cysts, which may be actively bleeding, torted [21] or hemorrhagic, tubal pathology such as a hydrosalpinx (Fig. 6) and other forms of pelvic inflammatory disease [22, 23]. Even the therapeutic management of various symptoms can depend on ultrasound findings such as size and location of tubo-ovarian abscesses, where treatment can be adapted depending on the findings. There have been cases in the literature where ultrasound-guided drainage of gynecological abscesses has shown a low complication rate [24] and where the use of POCUS has shown an immense reduction of clinical complications [25]. However, it should be kept in mind that a negative finding on POCUS is not proof of the absence of a possible severe diagnosis [29] (Fig. 7).

## Limitations of POCUS in OBGYN

One of the limitations of POCUS in OBGYN is the limited availability of transvaginal ultrasound probes. Although the transabdominal approach of scanning the pelvic region is used in the majority of POCUS examinations, the transvaginal (TV) approach is the preferred method of assessment in gynecology as well as in early pregnancy due to its superior results [26]. This is due to the use of a higher-frequency (7.5–12 MHz) transducer as well as the anatomical approach, which allows for closer contact with the organs of interest.

The current literature reveals a lack of knowledge regarding the application and methods of POCUS in OBGYN, although ultrasound is already widely used in the management of obstetric and gynecological emergencies. In countries where ultrasound is performed by radiologists or emergency physicians there is also a perceived lack of confidence by obstetric specialists in POCUS studies performed by emergency clinicians [33]. A survey investigated the confidence in scans done by internal medicine specialists and concludes that there is a paucity of highly qualified POCUS training in the field of emergency medicine [27].

Limited research has been performed in the field of POCUS among OBGYN specialists, particularly the level of proficiency and confidence in this modality and the efficiency of training sessions [28].

New residents in particular lack the theoretical and practical knowledge, which is required in daily clinical routine, even though there are the ones performing most of the initial physical and ultrasound examinations. The literature concludes that much practical training and a high frequency of supervision is necessary to master clinical skills [29]. The authors of these studies suggested that a better understanding of the training that is undergone by emergency physicians may inspire more trust and confidence in the results leading to a better credibility of the images and improve clinical decision-making processes [27].

## Need for POCUS programs in OBGYN?

The major issue concerning the practice for POCUS is its training within a clinical facility with trained personnel and equipment. It is acknowledged that guidelines are needed to establish the use of POCUS in a safe and effective way [30].

For the implementation of ultrasound training for OBGYN residents, a core curriculum is needed. This must contain clear learning objectives and well-defined outcome measures [31]. Such a curriculum was introduced by the European Board and College of Obstetrics and Gynaecology (EBCOG) within the last years [31]. Nevertheless, appropriate ultrasound and interpretation skills are needed by the examiner and, thus, carefully selected evaluation and teaching tools have to be assessed for these trainings [32]. A curriculum also has to be developed with local needs in mind, e.g., there are data suggesting that only one fifth of OBGYN residents are actually planning to perform or interpret obstetric ultrasound studies in their postgraduate training in the USA [29].

At the moment, there are no POCUS programs concerning OBGYN offered in Europe. The International Society for Ultrasound in Obstetrics and Gynecology (ISUOG) offers courses for healthcare practitioners and has a web-based learning platform (https://www.isuog.org/education.html), but this is not focused on point-of-care applications.

In Germany, training mainly consists of various DEGUM-approved courses especially teaching fetal duplex sonography and fetal echocardiography or IOTA-certification courses.

Current recommendations for the DEGUM Level I (for fetal ultrasound) study go beyond the minimum requirements of current maternity guidelines; they are much better suited to the current requirements of a second-trimester ultrasound. It enables qualified DEGUM Level I investigators to perform a ultrasound screening exam. These recommendations also define the requirements for advising pregnant women in the scope of ultrasound examinations, as well as the prerequisites for obtaining the DEGUM Level I qualification [32].

Besides the high-qualified educational programs an OBGYN POCUS program should focus on specific clinical indications such as the identification of intrauterine pregnancy, the diagnosis of acute pelvic pain as a common presenting complaint, ruptured ectopic pregnancy or pelvic inflammatory disease and tubo‐ovarian abscess. Thus, the benefit of establishing a POCUS program lies in its interdisciplinary expandability. In addition to the training of applications in the field of OBGYN, a POCUS course would also include the rapid sonographic diagnosis of pneumothorax, free fluid or the differential diagnosis of flank pain which is not taught in the current OBGYN programs in Germany [9].

In the field of emergency medicine, certain OBGYN applications are essential. In emergency clinical care, the determination of intrauterine pregnancy in the first trimester and the detection of fetal heartbeats are crucial and of great clinical importance. The diagnosis of an ovarian cyst or tubo-ovarian abscess is not essential as national and international needs assessments have shown [33].

In a third world setting, POCUS takes on a different perspective. Patients may have to travel long distances to access medical care. Many cannot afford the cost of transportation to a medical facility. There, POCUS could be used to identify high-risk patients who can be referred to regional hospitals for further management. Thus, a key feature of POCUS is that it is not a replacement for comprehensive ultrasound practice, but a focused ultrasound examination often performed under suboptimal conditions and with time limitations [28].

## Conclusion

POCUS is a clinical examination performed by the attending physician and is seen as an extension of the physical exam to gain information that influences the patient’s management and diagnosis. It is not a detailed ultrasound examination, and may provide only limited information, but can be used for the investigation of various, potentially severe gynecological and obstetrical symptoms.

The continuing development of portable ultrasound devices that are considerably cheaper than conventional ultrasound machines might facilitate access of more patients to ultrasound in the future. Standardization of training, ongoing education and assessment of practitioners performing POCUS is needed to establish its widespread uptake successfully. The main limitations for any implementation of POCUS trainings include access to tutors, time restriction and a lack of access to suitable and standardized POCUS equipment.
